# A comparison of plasma Tsukushi in adolescents with and without metabolic dysfunction‐associated steatotic liver disease

**DOI:** 10.14814/phy2.70988

**Published:** 2026-06-21

**Authors:** Sydni A. McNeese, Sadie A. Maltz, Youngsil Kim, April M. Teague, Sirish K. Palle, Jacob E. Friedman, Kevin R. Short

**Affiliations:** ^1^ Division of Diabetes and Endocrinology, Department of Pediatrics University of Oklahoma Health Campus Oklahoma City Oklahoma USA; ^2^ Division of Gastroenterology, Hepatology, and Nutrition, Department of Pediatrics University of Oklahoma Health Campus Oklahoma City Oklahoma USA; ^3^ Harold Hamm Diabetes Center University of Oklahoma Health Campus Oklahoma City Oklahoma USA; ^4^ Department of Biochemistry and Physiology University of Oklahoma Health Campus Oklahoma City Oklahoma USA

**Keywords:** adolescents, biomarker, hepatokine, obesity

## Abstract

Metabolic dysfunction‐associated steatotic liver disease (MASLD) is the most common liver disease in children and adolescents. Assessment of circulating biomarkers is needed to understand the underlying pathology, disease severity, and treatment options. The secreted hepatokine Tsukushi (TSK) is involved in lipid and carbohydrate metabolism. TSK is increased in adults with MASLD but has yet to be reported in adolescents. We hypothesized that plasma TSK is increased in adolescents with MASLD compared to peers without MASLD classified as obese (Ob) or normal weight (NW). We tested adolescents 10–20 years old with obesity and biopsy‐confirmed MASLD (17F, 46 M), and controls without MASLD with either Ob (16F, 16 M) or NW (15F, 20 M). Fasting plasma TSK concentration was 27% higher (*p* < 0.05) in the MASLD group (median [interquartile range]: 188 [120, 418] ng/mL) than the NW group (148 [80, 241]), while the Ob group was intermediate (165 [84, 466]) and not different from the other two groups. TSK did not differ across histological grades of steatosis or fibrosis. Although TSK was increased in the MASLD group compared to NW controls, the lack of difference in TSK between the Ob and MASLD groups limits its value as a potential biomarker for MASLD.

## 1 INTRODUCTION

Pediatric metabolic dysfunction‐associated steatotic liver disease (MASLD), defined by fat accumulation in the liver that can be accompanied by inflammation, hepatocyte ballooning, and fibrosis, is present in about 36% of children and adolescents with obesity (Shaunak et al., 2021). The disease can progress to metabolic dysfunction‐associated steatohepatitis (MASH) and patients with MASLD are at increased risk of developing type 2 diabetes (Nobili et al., 2019; Vos et al., 2017).

Compared to MASLD in adults, pediatric MASLD often differs in histology and can have a more rapid disease progression (Kleiner & Makhlouf, 2016; Shaunak et al., 2021). Children are more likely to have liver steatosis and fibrosis in portal regions than adults and this may be associated with the presence of specific genetic markers (Hudert et al., 2019; Kleiner & Makhlouf, 2016). The current gold standard for pediatric MASLD diagnosis is liver biopsy, but there are ongoing efforts to identify non‐invasive biomarkers of the disease to track changes over time (Martinou et al., 2022). Several hepatokines have been reported in animal models and studies of adults, with some demonstrating increases in response to over‐feeding and regulation of hepatic insulin signaling and steatosis (e.g., angiopoietin‐like proteins), and others (e.g., fetuins A and B) demonstrating communication with other organs (Kim et al., 2021). Some biomarkers are less useful in pediatric patients than adults. For example, the epitope of pro‐peptide of type III collagen formation (PRO‐C3) predicts hepatic fibrosis stage in adults with MASLD but is less reliable in pediatric MASLD due to its variation with age and pubertal status and its association with bone turnover, which is higher in developing adolescents than adults (Cohen et al., 2021). This underscores the need to carefully evaluate proposed MASLD biomarkers in adolescent populations.

Tsukushi (TSK) is a recently‐described small leucine‐rich proteoglycan that is secreted from the liver and increases in the liver and circulation when liver steatosis is elevated in mice and adult humans (Furuhashi et al., 2022; Grander et al., 2020; Lam et al., 2024; Mouchiroud, Camiré, et al., 2019; Xiong et al., 2019). In mice on a MASH‐promoting diet, whole body knock‐out of TSK resulted in lower accrual of body fat and smaller livers compared to wild‐type mice (Xiong et al., 2019). Further, hepatic TSK protein abundance returned to normal when mice on the MASH‐promoting diet were switched to normal chow, suggesting that TSK plays a role in the pathophysiology of lipid metabolism. In adults with MASLD, serum TSK was positively associated with liver fibrosis, independent of type 2 diabetes status (Lam et al., 2024). Another study showed that serum TSK was elevated in adults with metabolic syndrome compared to controls (Li et al., 2023). However, many patients in both of those human studies had multiple co‐morbidities, so it is unclear whether elevated TSK concentration was attributable specifically to the presence of MASLD or exacerbated by other conditions. Furuhashi et al. (Furuhashi et al., 2022) reported in a large cohort of adults who did not use medications for co‐morbidities like diabetes, hypertension, or other cardiometabolic conditions, that TSK was positively correlated with insulin resistance and a fatty liver index (derived from values for body mass index, waist circumference, triglycerides, and γ‐glutamyl transferase), but was not significantly correlated with individual liver enzymes. Thus, while there is some support for TSK as a marker of liver health in adults, there remains a need to further confirm its role. Importantly, circulating TSK has not yet been reported in a pediatric population.

The goal of this study was to test the hypothesis that plasma TSK concentration is elevated in adolescents with obesity and biopsy‐confirmed MASLD compared to peers without MASLD who had either obesity or normal weight.

## 2 MATERIALS AND METHODS

### 2.1 Ethics statement

This is a secondary analysis of samples and data from three consecutive studies designed to measure metabolic, physiologic, and biochemical differences among adolescents with and without MASLD. Each study had the same set of inclusion and exclusion criteria and the same set of core tests and procedures that could be used for the current analyses (15–17). Participant testing and blood sample collection for the current analyses occurred from February 2019 to July 2024. Some of the participants and their descriptive characteristics in the current study have appeared in prior publications (Bade et al., 2025; Bartlett et al., 2025; Bays et al., 2025), but the primary outcome on circulating TSK concentration has not. The third study in this series was registered at clinicaltrials.gov (NCT05430178) in June 2022. Informed written consent and/or assent was obtained from all participants and their parent/guardians following guidelines of the University of Oklahoma Health Campus Institutional Review Board for Human Subjects, which approved the studies. The study was conducted in accord with the Declaration of Helsinki.

### 2.2 Participants

All study participants were 10.2–20.7 years old. We compared three groups: (1) a control group with normal weight and without MASLD (NW), (2) a control group with obesity and without MASLD (Ob), and (3) a group with obesity and MASLD confirmed by liver biopsy. Normal weight was classified as a body mass index (BMI) between the 5th and 85th percentile for sex and age based on pediatric growth charts from the United States Centers for Disease Control and Prevention (Kuczmarski et al., 2002). Obesity was defined as a BMI ≥95th percentile for sex and age on the same charts (Kuczmarski et al., 2002). Patients enrolled into the MASLD group had persistently elevated serum alanine aminotransferase (ALT), prior ultrasound findings consistent with MASLD, and biopsy findings confirming MASLD. They were excluded if they were found to have a liver disorder other than MASLD. Control group participants had no clinical evidence of liver disease, including an ALT value <47 U/L (the healthy reference range for our laboratory), upper abdominal pain, severe fatigue, or unexplained weight loss or weakness. Exclusion criteria for all participants included cardiovascular, endocrine, or renal conditions that could interfere with liver function, or physical conditions that limited ability to exercise. Additional exclusion factors were use of medications that could impact physical or metabolic function, and current or recent use of tobacco, cannabis, alcohol, or illicit drugs. We recognize that participants ≥18 years old are often referred to as young adults based on legal definition, but the World Health Organization defines adolescence as 10–19 years old (World_Health_Organization, 2026). We set the upper age limit for inclusion at 20 years old as there are several patients in our center who remain in the care of a pediatrician until that age. There were 2 males in the NW group and 1 male in the Ob group who were >19 years old but for simplicity we used the term adolescents throughout the paper.

### 2.3 Protocol

After an overnight fast, study participants arrived in the laboratory at 8:00 am and completed a venous blood sample collection. After centrifugation, aliquots of serum and plasma were stored at −80°C until analysis. Height and body mass were measured with a calibrated stadiometer and a balance, respectively, and used to calculate BMI and BMI *z*‐score (Kuczmarski et al., 2002). Total body fat and lean soft tissue masses were measured with a dual energy X‐ray absorptiometer (GE/Lunar iDXA, GE‐Healthcare, Fairfield, CT), as reported previously (Short et al., 2018).

Liver biopsies were performed in patients with MASLD as part of routine clinical care. Two certified pathologists performed standard histopathological evaluation of all biopsies, assessing the severity of steatosis, inflammation, and fibrosis to determine the MASLD Activity Score (MAS), and fibrosis stage using a standardized method (Africa et al., 2018; Kleiner et al., 2005).

### 2.4 Biochemical analyses

Glycated hemoglobin (HbA1c) was measured on whole blood using a Siemens DCA Vantage analyzer (Tarrytown, NY). Glucose, total cholesterol, HDL‐cholesterol, triglycerides, ALT, and aspartate aminotransferase (AST) were measured in blood collected in lithium‐heparin coated tubes using a Piccolo Xpress Chemistry Analyzer (Abbott Point of Care, Princeton, NJ). LDL‐cholesterol was calculated using a published equation (Sampson et al., 2020). Serum insulin was measured with an enzyme‐linked immunosorbent assay (ELISA) from Alpco (#80‐INSHU‐CH01, Salem, NH). Insulin resistance was calculated from fasting glucose and insulin values using the revised integrated homeostatic model of assessment (iHOMA2‐IR) (Hill et al., 2013). Tsukushi was measured in duplicate in EDTA plasma using an ELISA from RayBiotech (#ELH‐TSKU, Peachtree Corners, GA). The manufacturer states that the minimal detectable concentration of human TSK is 0.63 ng/mL and that the assay does not have cross‐reactivity with 38 proteins, including several found in the liver and/or circulation. The intra‐ and inter‐assay coefficients of variability reported by the manufacturer are <10 and <12%, respectively. In our hands those values were both <5%. To minimize batch effects, each assay run included samples from each of the three study groups.

### 2.5 Data analysis

Since this was an exploratory, secondary analysis and because there were no prior data on TSK in adolescents, we did not calculate a required sample size prior to conducting the current analyses. To avoid bias we used all participants who enrolled during the stated time period, met the inclusion and exclusion criteria, and had available samples and data. For comparisons among groups, a Brown‐Forsythe test was used to determine equality of variances. Group comparisons were performed using either analysis of variance and Tukey's post‐hoc test for multiple comparisons, or a Kruskal–Wallis test and Dunn's tests for post‐hoc comparisons, as appropriate. A two‐way analysis of variance was used to determine the biological effect of sex among groups. Chi‐square tests were used to assess proportional differences in males and females among groups. Spearman's correlations were used to determine the strength of association between plasma TSK and other participant characteristics. All analyses and figures were completed with GraphPad Prism 10.6. A *p*‐value less than 0.05 was considered statistically significant for all tests.

## 3 RESULTS

### 3.1 Participant characteristics

Descriptive characteristics for the NW, Ob, and MASLD groups are shown in Table 1. There were 29 more males than females in the MASLD group, but the Chi square test for proportional differences of males and females among groups did not reach statistical significance. The MASLD group was younger than both the NW and Ob control groups. By design, the Ob and MASLD groups were heavier and had significantly higher BMI (kg/m^2^) and BMI *z*‐scores than the NW group. The MASLD group also had a higher median BMI *z*‐score than the Ob group. Body fat percentage was higher in the MASLD and Ob groups than in the NW group. The MASLD group had higher fasting glucose concentration than the NW and Ob groups but did not differ from the Ob group for fasting insulin concentration and insulin resistance. Both the Ob and MASLD groups had higher insulin concentration and insulin resistance than the NW group. The groups did not differ in total cholesterol or LDL‐C concentrations, but the MASLD group had lower HDL‐C than the Ob and NW groups. The Ob group also had lower HDL‐C than the NW group. The MASLD group had higher values for triglycerides, ALT, and AST compared to both the Ob and NW groups.

**TABLE 1:** Participant characteristics.

	NW control	Ob control	MASLD	*p*‐value
Sex (M:F)	20:15	16:16	46:17	0.062
Age (years)	15.5 ± 2.5	15.8 ± 2.5	14.1 ± 2.3^a,b^	0.001
Height (cm)	165.6 ± 11.4	165.8 ± 10.8	165.5 ± 11.3	0.982
Weight (kg)	57.5 [50.8, 61.5]	86.8 [71.9, 101.8]^a^	91.5 [78.9, 107.5]^a^	<0.001
BMI z‐score	0.24 [−0.33, 0.56]	1.97 [1.85, 2.19]^a^	2.30 [2.04, 2.52]^a,b^	<0.001
Body fat (%)	29.5 ± 7.5	42.9 ± 7.0^a^	46.5 ± 5.6^a^	<0.001
Lean soft tissue mass (kg)	35.6 ± 8.9	45.8 ± 12.1^a^	45.2 ± 11.3^a^	<0.001
HbA1c (%)	5.4 ± 0.3	5.4 ± 0.4	5.6 ± 0.6	0.059
Glucose (mmol/l)	4.6 [4.2, 5.1]	4.4 [4.1, 5.2]	5.4 [4.8, 6.2]^a,b^	<0.001
Insulin (pmol/l)	49.2 [27.6, 72.6]	115.8 [69.6, 159.6]^a^	128.4 [69.0, 255.6]^a^	<0.001
iHOMA2‐IR (units)	0.84 [0.52, 1.36]	2.07 [1.27, 2.54]^a^	2.43 [1.35, 4.68]^a^	<0.001
Total cholesterol (mmol/l)	4.07 ± 0.87	3.95 ± 1.06	4.17 ± 1.00	0.579
HDL‐C (mmol/l)	1.40 [1.16, 1.71]	1.19 [1.01, 1.45]^a^	1.03 [0.90, 1.19]^a,b^	<0.001
LDL‐C (mmol/)	2.30 ± 0.78	2.34 ± 0.89	2.52 ± 0.89	0.702
Triglycerides (mmol/l)	0.87 [0.69, 1.13]	1.18 [0.72, 1.46]	1.94 [1.34, 2.70]^a,b^	<0.001
ALT (mU/ml)	19 [15, 20]	25 [15, 38]	85 [56, 154]^a,b^	<0.001
AST (mU/ml)	22 [24, 33]	28 [20, 36]	59 [42, 92]^a,b^	<0.001

*Note*: Results presented as participant counts for sex, mean ± standard deviation for normally‐distributed variables, and median [upper, lower interquartile range] for non‐normally‐distributed variables. *p*‐values shown for the main effect of group assessed with a one‐way analysis of variance or Kruskal–Wallis test, except for sex distribution, where Chi‐square tests were used. Post‐hoc differences between groups are designated as ^a^different from NW, *p* < 0.05; ^b^different from Ob, *p* < 0.05 after controlling for multiple comparisons.

Abbreviations: ALT, alanine aminotransferase; AST, aspartate aminotransferase; BMI, body mass index; HbA1c, glycated hemoglobin; HDL‐C, high‐density lipoprotein cholesterol; iHOMA2‐IR, interactive homeostatic model of assessment for insulin resistance; LDL‐C, low‐density lipoprotein cholesterol.

The distribution of liver histological scores for steatosis, MASLD Activity Score, and fibrosis for the MASLD group are shown in Table 2. Ninety percent of the MASLD patients had steatosis grade 2 or 3. Only 3 out of 63 patients had MASLD Activity Scores that classified them as not having MASH. The fibrosis stage was absent or mild (0–1) in 38% of MALSD patients, while 33% had periportal fibrosis (1c). Only 8 patients (13%) had advanced (stage 3 or 4) fibrosis.

**TABLE 2:** Distribution of liver histology results in patients with MASLD.

Histological feature and grade/stage	Number of patients
Steatosis	
0, <5%	0
1, 5%–33%	6
2, >33%–66%	38
3, >66%	19
MASLD Activity Score	
0, not MASH	0
1, not MASH	0
2, not MASH	3
3, borderline MASH	9
4, MASH	31
5, MASH	19
6, MASH	1
Fibrosis	
0, none	10
1a, mild perisinusoidal	14
1b, moderate perisinusoidal	1
1c, portal/periportal	21
2, perisinusoidal and portal/periportal	9
3, bridging	7
4, cirrhosis	1

*Note*: MASLD Activity Score is the sum of the steatosis, lobular inflammation, and ballooning sub‐scores. The MAS range is 0–8, but no participants scored above 6.

### 3.2 Plasma Tsukushi concentration and association with participant characteristics

**FIGURE 1:**
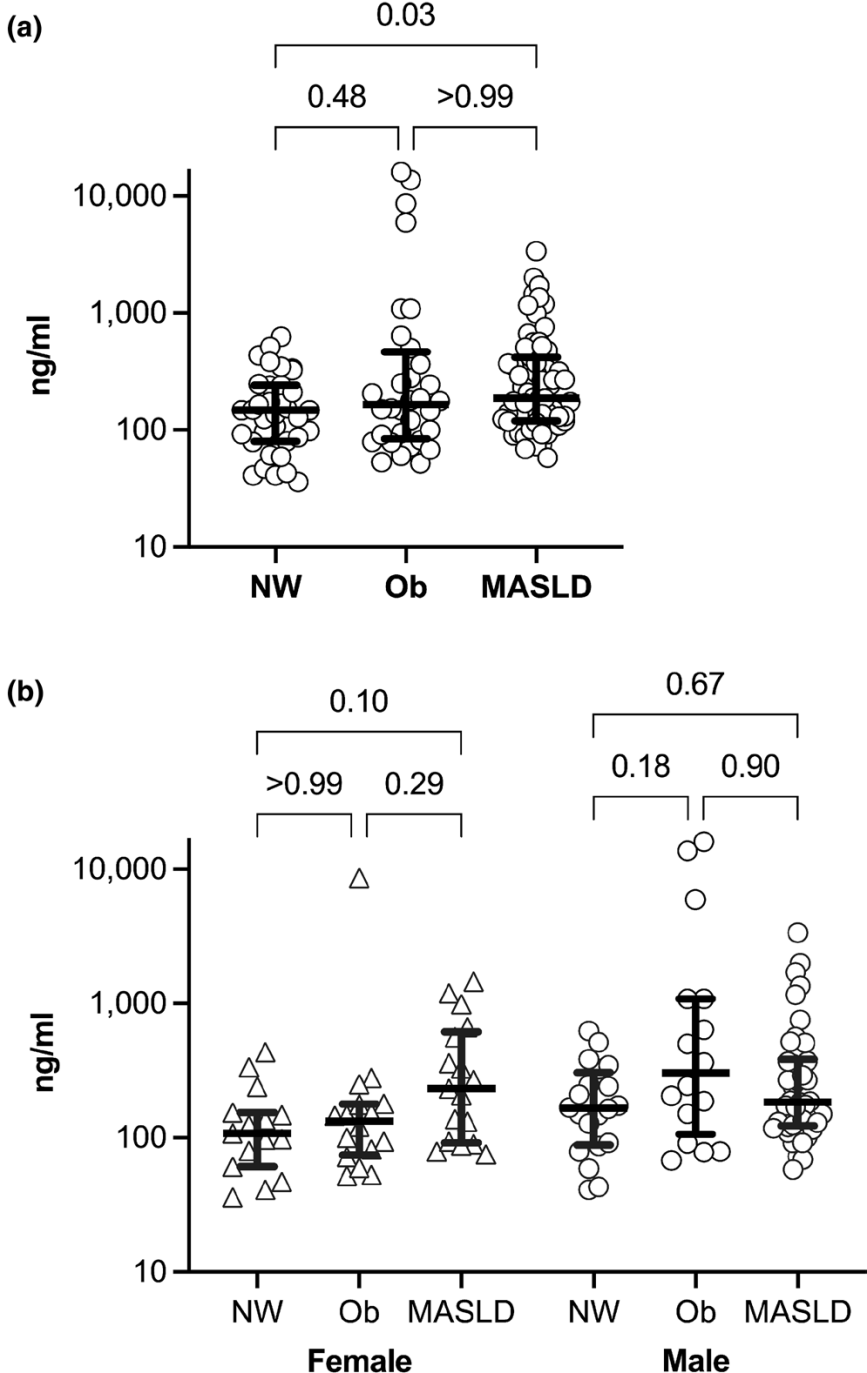
(a) Plasma concentration of Tsukushi in control groups without metabolic dysfunction‐associated steatotic liver disease (MASLD) and with normal weight (NW) or obesity (Ob), respectively, and participants with obesity and MASLD. (b) Groups subdivided into female and male participants. Y‐axes are presented as log‐10 scale. *p*‐values for pairwise comparisons among groups are from Dunn's tests (a) and Tukey's tests (b), respectively, corrected for multiple comparisons.

The median value for plasma TSK was 27% higher in the MASLD group than the NW group, *p* < 0.05 (Figure 1). There were no other significant differences among groups as the Ob group was intermediate and there was a wide range of values within all three groups. Figure 1 also demonstrates that there were no significant group differences in TSK in females or males when considered separately, although in females, the median value for the MASLD group was approximately double that of the NW and Ob groups. Plasma TSK concentration did not differ significantly between males and females, either for the whole cohort (main effect, *p* = 0.08), or for within‐group comparisons (adjusted *p*‐values for male–female comparisons were >0.99, 0.08, and >0.99 for the NW, Ob, and MASLD groups, respectively). Notably, these results were influenced by four outliers in the Ob group (1 female, 3 male). When those results were removed, the main effect of sex was reduced for the whole cohort (*p* = 0.22) and within the Ob group (*p* = 0.69).

**FIGURE 2:**
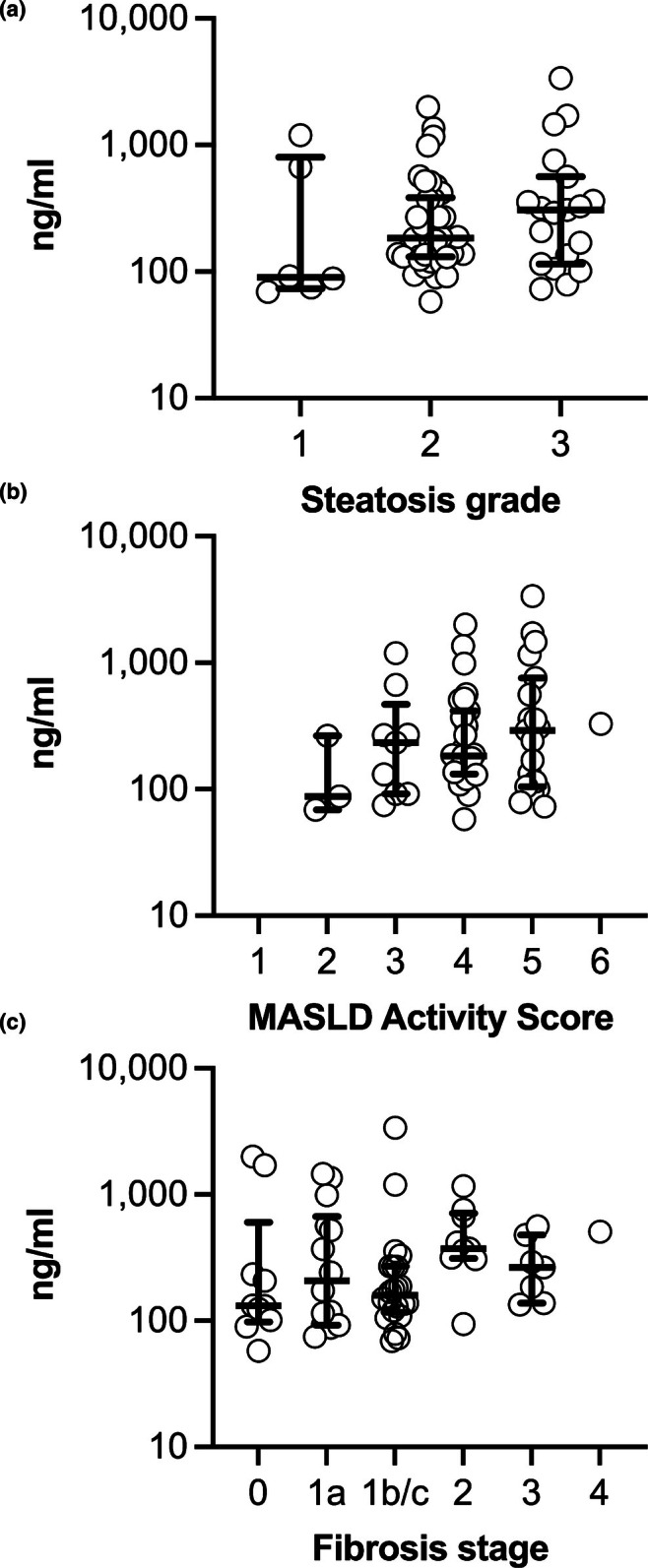
Plasma Tsukushi concentration for MASLD patients across the range of liver histology scores. (a) Steatosis grade (full range = 0–3); (b) MASLD Activity Score (full range = 0–8); (c) Fibrosis stage (full range = 0–4). Fibrosis stages 1b and 1c are combined since there was only 1 patient with stage 1b. Y‐axes are presented as log‐10 scale. There were no statistically significant differences for TSK across histology values.

Table 3 shows the Spearman correlations between plasma TSK and the participant characteristics. While there were 5 significant correlations for the whole cohort (BMI‐z, lean mass, HDL, ALT, and AST), the magnitudes of association were generally low. Insulin and insulin resistance were positively correlated with TSK in the NW group, but there were no significant relationships in the Ob or MASLD groups or for the whole cohort. Additionally, plasma TSK did not significantly vary across the range of liver steatosis, fibrosis, or MASLD Activity Score values within the MASLD group (Figure 2). None of the reported TSK results presented were significantly altered by the inclusion of the 3 participants (2 NW males, 1 Ob male) who were >19 years old.

**TABLE 3:** Spearman correlations between Tsukushi and selected variables.

	All	NW	Ob	MASLD
Age	0.040	−0.052	−0.035	0.245
BMI‐z	0.173*	0.181	−0.213	0.015
Body fat (%)	0.082	0.008	−0.264	−0.047
Lean soft tissue mass (kg)	0.196*	0.082	−0.309	0.173
Glucose (mg/dl)	0.113	0.169	−0.191	0.133
Insulin (μIU/ml)	0.072	0.355*	−0.254	−0.053
iHOMA2‐IR (units)	0.083	0.372*	−0.233	−0.044
Total cholesterol (mg/dl)	0.099	0.152	−0.192	0.205
HDL‐C (mg/dl)	−0.152	0.003	−0.043	−0.033
TG (mg/dl)	0.124	0.195	−0.331	0.097
ALT (mU/ml)	0.239**	0.126	0.119	0.085
AST (mU/ml)	0.244**	0.186	0.140	0.136

* *p* < 0.05.

** *p* < 0.01.

## 4 DISCUSSION

We found that plasma TSK was significantly higher in adolescents with MASLD compared to the NW control group but not different from the Ob control group. This difference was driven by larger nominal (though not statistically significant) group differences in females. Across all participants, higher serum TSK was weakly associated with reduced HDL‐C, implicating a role in systemic lipid dysregulation.

### 4.1 Group differences

We did not find a difference in plasma TSK between males and females overall but TSK in females with MASLD group tended be higher than the females in the NW and Ob groups, while between‐group differences were absent in males. A prior study of mice reported that females had lower liver expression of TSK than males and that TSK increased in females following ovariectomy (Meda et al., 2022). That outcome was interpreted as one of the potential mechanisms through which estrogen is thought to protect against the development of MASLD (Cherubini et al., 2024). The higher number of males than females with MASLD in our study reflects the higher propensity for adolescent males to develop MASLD compared to females of similar age (Anderson et al., 2015; Shakir et al., 2018). Sex differences have not been reported in most of the prior human studies on TSK. Furuhashi et al. (Furuhashi et al., 2022) found that plasma TSK did not differ between men and women who were part of a general population cohort, but their mean age was 57 years old and the number of women who were postmenopausal was not stated.

### 4.2 Association between TSK and other variables

We found that plasma TSK was generally weakly correlated with some of the descriptive characteristics of the participants, including lean soft tissue mass, BMI‐z, and common blood biochemistry values. Counter to expectations, there was no variance in TSK across grades and stages of liver histology. In contrast to our findings, Lam et al. (Lam et al., 2024) reported in a large cohort of adults (mean age 60 years old) that plasma TSK was positively correlated with estimates of liver steatosis and stiffness measured with transient elastography. However, the magnitudes of those relationships were fairly low with correlation coefficients in the range of 0.12–0.34 among subgroups with and without type 2 diabetes. Studies of adults and mouse models have provided some evidence that TSK regulates cholesterol and severity of steatosis. A study of mice using a gain‐ and loss‐of‐function approach found that TSK lowers cholesterol efflux capacity, reduces circulating HDL‐C, and reduces the cholesterol‐to‐bile acid conversion in the liver (Mouchiroud, Camire, et al., 2019). Plasma TSK was recently reported to be inversely correlated with HDL‐C and cholesterol efflux capacity in adults with MASLD (Lam et al., 2025) while two other studies reported a small but significant inverse correlation between plasma TSK and either total cholesterol or HDL‐C concentrations (Furuhashi et al., 2022; Li et al., 2023). Li et al. (Li et al., 2021) also reported that plasma TSK was elevated in adults with either high triglycerides (TSK was 16% higher) or low HDL‐C (TSK was 33% higher). We found a small but significant inverse correlation between TSK and HDL‐C for the whole cohort, but that relationship was not present within any of the study groups when considered separately and there was not a significant correlation with total cholesterol. Thus, in our adolescent cohort, the association between circulating TSK and lipids appears to be weaker that what has been reported in adults or animals studies. This may be because none of our participants had comorbidities like type 2 diabetes, used tobacco, consumed alcohol, or used medications for hypertension or dyslipidemia, which are more common in studies of adults (Furuhashi et al., 2022; Lam et al., 2024, 2025; Li et al., 2021).

### 4.3 Comparison to studies of adults

The median TSK concentrations in our study were higher (group medians from 148 to 188 ng/mL, overall range = 36 to 16,023 ng/mL), than reported in three studies of adults that used the same ELISA (Furuhashi et al., 2022; Lam et al., 2024, 2025). In two studies conducted by Lam et al. (Lam et al., 2024, 2025) on adults with MASLD with and without type 2 diabetes and an average age of ~58 years old, median serum TSK concentrations were approximately 100 ng/mL (range ~12 to 1780 ng/mL) while Furuhashi et al. (Furuhashi et al., 2022) reported that median plasma TSK was 28 ng/mL (range ~5 to 1000 ng/mL) in adults with an average age of 57 years old. Similar to our finding, in each of those prior studies, there was a wide range of TSK values, with several high outliers, but the reason for that variance is not yet clear.

### 4.4 Strengths and limitations

One of the strengths of the current study was the inclusion of pediatric patients with biopsy‐confirmed MASLD. That allowed us to test whether plasma TSK varied with the severity of liver histology scores. Although there were no trends for variance in TSK across histology scores, the values for our MASLD group did not span the full potential range for histological features. Samples with low or no steatosis were not available, for example, since liver biopsies from healthy adolescents with Ob or NW are difficult to acquire. Similarly, pediatric patients with MASLD typically do not have more advanced fibrosis or MASLD Activity Scores. However, the patients in the current study are reflective of our clinical practice (Shakir et al., 2018), which reinforces our finding that the lack of difference in TSK between the MASLD and Ob groups limits the practical use of TSK as a biomarker for MASLD.

A limitation of our study was that we did not perform dietary assessments, which could be used to determine if habitual intake of specific dietary components contributes to the variation in plasma TSK. We also relied on liver enzyme measurements and standard clinical assessments to assign participants to the control groups. We did not have estimates of liver steatosis or fibrosis from imaging modalities such as transient elastography or magnetic resonance imaging on the whole cohort, which could be useful to determine whether TSK varies across the range of those variables. Lastly, we did not measure TSK abundance in the liver, which could have been useful to determine the relationship between tissue and plasma content. Liver biopsies in pediatric patients are small and prioritized for clinical evaluation, limiting the ability to measure TSK in tissue.

## 5 CONCLUSIONS

Plasma TSK was elevated in adolescents with MASLD compared to NW controls but not different from Ob controls. Although TSK was significantly correlated with some descriptive variables, those relationships were generally weak, and plasma TSK did not vary among histological grades and stages in patients with MASLD. Thus, our findings do not support a clear role of TSK in the development or progression of MASLD in adolescents.

## AUTHOR CONTRIBUTIONS

**Sydni A. McNeese:** Conceptualization; data curation; formal analysis; investigation; visualization. **Sadie A. Maltz:** Data curation; investigation; methodology. **Youngsil Kim:** Data curation; investigation; supervision. **April M. Teague:** Data curation; supervision. **Sirish K. Palle:** Conceptualization; funding acquisition; investigation. **Jacob E. Friedman:** Conceptualization; funding acquisition. **Kevin R. Short:** Conceptualization; formal analysis; funding acquisition; investigation; project administration; supervision.

## FUNDING INFORMATION

Research reported in this publication was supported by the National Institutes of Health through grants from the National Institute of Diabetes, Digestive, and Kidney Diseases (R01DK129656) and the National Institute of General Medical Sciences (U54GM104938). The content is solely the responsibility of the authors and does not necessarily represent the official views of the National Institutes of Health. Additional support was provided by the Presbyterian Health Foundation, the Oklahoma Children's Health Foundation, and the Pediatric Metabolic Research Program.

## CONFLICT OF INTEREST STATEMENT

S.K.P. is on the advisory board for Mirum Pharmaceuticals, but that work is unrelated to the topic of this study. All other authors have nothing to declare.

## ETHICS STATEMENT

All procedures involving human participants were reviewed and approved by the University of Oklahoma Health Campus Institutional Review Board for Human Subjects. The study was conducted in accordance with the Declaration of Helsinki. All participants and their parent/guardians provided their informed written consent and/or assent prior to participation.

## CLINICAL TRIAL REGISTRATION

A subset of participants included in this analysis was enrolled into a protocol that was registered at clinicaltrials.gov (NCT05430178) in June 2022.

## Data Availability

Data will be made available upon reasonable request.
